# Assessment of the effect of 21-day head-down bed rest on the cardiovascular system by blood protein composition

**DOI:** 10.3389/fphys.2024.1375929

**Published:** 2024-06-20

**Authors:** Daria N. Kashirina, Ludmila Kh. Pastushkova, Anna G. Goncharova, Irina M. Larina

**Affiliations:** SSC RF–Institute of Biomedical Problems of the Russian Academy of Sciences, Moscow, Russia

**Keywords:** head-down bed rest, mass spectrometry, blood, cardiovascular system, retinol-binding protein 4

## Abstract

Head-down bed rest (HDBR) is one of the models of the physiological effects of weightlessness used, among other things, to assess the effect of hypokinesia on the physiological systems of the human body and, first of all, on the cardiovascular system. The aim of the work was to study the effect of 21 days of HDBR factors on the cardiovascular system based on blood proteomic profile data. It was revealed that HDBR conditions led to an increase in the levels of proteins of the complement and the coagulation cascade systems, platelet degranulation, fibrinolysis, acute phase proteins, post-translational modification of proteins, retinol-binding protein 4 (RBP4), apolipoprotein B, which are associated with cardiovascular diseases, and other proteins that affect the functions of endothelial cells. Blood levels of proteins involved in cytoskeletal remodelling, oxygen transport, heme catabolism, etc. have been shown to decrease during HDBR.

## 1 Introduction

Head-down bed rest (HDBR) is not only one of the models of the physiological effects for weightlessness, but also for hypodynamia, which can be considered as an inevitable companion of scientific and technological progress, accompanied both by a significant reduction in the amount of physical labour and by a characteristic lifestyle that has become more and more widespread in recent times. The modern lifestyle leads to metabolic changes and a deterioration in vascular function, which can ultimately have a negative impact on health. As noted, hypokinesia (a sharp reduction in motor activity, a decrease in muscle work), along with the spread of a Western diet, is one of the most serious causes of the emergence and threateningly rapid spread of cardiovascular and neurological diseases, as well as diseases associated with disorders of the gastrointestinal tract, endocrine glands and other organs and systems (Davies et al., 2017).

It is known that exposure to HDBR is associated with the development of compensatory adaptive responses involving the cardiovascular, endocrine, central, and peripheral nervous systems (Boutouyrie et al., 2022; Buoite et al., 2021; Blanc et al., 2000). During the acute phase of HDBR, participants experience a decrease in circulating plasma volume and a movement of extracellular fluid and blood volumes in a cranial direction (Millet et al., 2001). The responses of the cardiovascular system to blood redistribution are currently being actively investigated. In a 21-day HDBR experiment, a decrease in left ventricular mass and diastolic volume was observed (Greaves et al., 2019). A decrease in the heart volume leads to lower cardiac output (decrease in systolic and minute blood volume). In this case, the heart rate increases both at rest and during exercise. The redistribution of vasoconstrictor and pressor influences in the vessels of different regions of the body observed during HDBR (Baranov et al., 2016) also affects systemic hemodynamics and cardiovascular system (CVS) function. It has recently been shown that HDBR causes a significant increase in aortic stiffness, the parameters of which did not fully recover 1 month after the end of the experiment, and significant changes in the size of the thoracic aorta and the muscular arteries of the lower body were also revealed (Boutouyrie et al., 2022).

It has been shown that carbohydrate metabolism is also altered in HDBR: insulin secretion increases and glucose tolerance decreases (Blanc et al., 2000; Afonin, 1989). The development of insulin resistance can lead to endothelial dysfunction, which does not allow the vessels to function effectively and is dangerous because of the possibility of developing cardiovascular disease. Therefore, early prevention of insulin resistance and endothelial dysfunction and the search for means and methods to restore vascular function remain the most promising areas in the prevention and treatment of cardiovascular disease.

It is well known that prolonged bed rest causes muscle wasting. Proteomic methods have shown that HDBR leads to changes in the levels and phosphorylation of structural and metabolic muscle proteins (Dillon et al., 2019). Inhibition of muscle focal contact proteins and an increase in the levels of antioxidant response pathway proteins in slow (but not fast) muscle fibres were also observed. HDBR activated markers of neuromuscular damage (Murgia et al., 2022). Proteomic analysis of urine proteins from volunteers participating in HDBR suggested that blood coagulation processes such as fibrinolysis and regulation of cell adhesion were aimed at maintaining homeostasis and were activated in the early period of the experiment. Under the influence of a complex of factors of long-term bed rest, proteolysis and metabolism of oligosaccharides were activated (Pastushkova et al., 2017).

In recent years, proteomics methods based on mass spectrometry have been widely used to study the protein composition of body fluids from healthy individuals in order to analyse biological processes in the body. Understanding protein expression is the key to deciphering the mechanisms of action of various factors and, ultimately, to finding effective countermeasures to prevent adverse changes. The study of the composition of blood proteins functionally associated with the cardiovascular system by proteomic methods will contribute to the understanding of the mechanisms underlying cardiovascular changes under the influence of hypokinesia conditions. This knowledge may also help to understand the pathophysiology of common cardiovascular diseases. In this work, the protein composition of the blood plasma of volunteers was studied using proteomic methods to elucidate the molecular mechanisms of the changes that occurred during the 21-day HDBR.

## 2 Methods

### 2.1 Design of HDBR

The 21-day HDBR study involved 6 healthy men aged between 24 and 41 years. Each participant voluntarily signed an Informed Consent form after the potential risks, benefits and nature of the study were explained. All subjects were confirmed by a committee of medical experts to be in good health, free of chronic disease, and with a normal body mass index. All participants in the experiment were placed in a head-down position on a bed with an inclination angle of -6° for 21 days. Venous blood was collected in Vacuette EDTA tubes on an empty stomach 6 days before the start of the experiment and on the 21st day of HDBR.

### 2.2 Preparation of plasma samples for proteomic analysis

Plasma samples were purified from major proteins using Top 12 columns (Pierce Chemical Co., United States of America) and then standard sample preparation, including the steps of reduction, alkylation, precipitation, and trypsinolysis, was performed (Kashirina et al., 2020). The resulted tryptic peptide mixture was analyzed using liquid chromatography–mass spectrometry method based on a nano-HPLC Dionex Ultimate3000 system (Thermo Fisher Scientific, United States of America) and a timsTOF Pro (Bruker Daltonics, United States of America) mass spectrometer. Peptides were separated using a packed emitter column (C18, 25 cm × 75 μm x 1.6 µm) (Ion Optics, Parkville, Australia) at a flow rate of 400 nL/min by gradient elution with 4%–90% phase B during 40 min. Mobile phase A consisted of 0.1% formic acid in water and mobile phase B consisted of 0.1% formic acid in acetonitrile.

Mass spectrometric analysis was performed using the Parallel Accumulation - Serial Fragmentation (PASEF) acquisition method (Meier et al., 2018). An electrospray ionization (ESI) source was operated at a capillary voltage of 1500 V, an end plate bias of 500 V and 3.0 L/min of dry gas at temperature of 180°C. The measurements were carried out in the m/z range from 100 to 1700 Th. The ion mobility was in the range from 0.60 to 1.60 V s/cm^2^. The total cycle time was 1.88 s and the number of PASEF MS/MS scans was set to 10.

### 2.3 Data analysis

The resulting LC-MS/MS data were analyzed using PEAKS Studio 8.5. Only proteins identified by at least 2 peptides, one of which was specific for a particular protein, were subjected to further analysis. The given limiting parameters were as follows: parent mass error tolerance - 20 ppm; fragment mass error tolerance - 0.03 Da; enzyme - trypsin; the maximum number of missed cleavages - 3; fixed modifications - carbamidomethyl C); variable modifications - oxidation M), acetylation (N-terminus). The false positive rate (FDR) threshold was set to 0.01.

All the LC-MS/MS results have been deposited to the ProteomeXchange Consortium via the PRIDE (Vizcaino et al., 2016) partner repository with the dataset identifier PXD053149.

### 2.4 Statistical and bioinformatic methods of data processing

Statistical analysis was carried out using the Statistica 12 program. The homogeneity of sample variances was assessed using the Levene test. The normality of data distribution in each group was determined using the Shapiro-Wilk test. In the absence of normal distribution and equal variances, the Mann-Whitney test was used, which is a nonparametric alternative to the *t*-test.

Molecular functions and biological processes enriched with the identified proteins were determined using the STRING web resource (https://string-db.org), and the search for links with processes associated with the cardiovascular system was performed using the ANDSystem program (http://www-bionet.sscc.ru/and/cell).

## 3 Results

**FIGURE 1:**
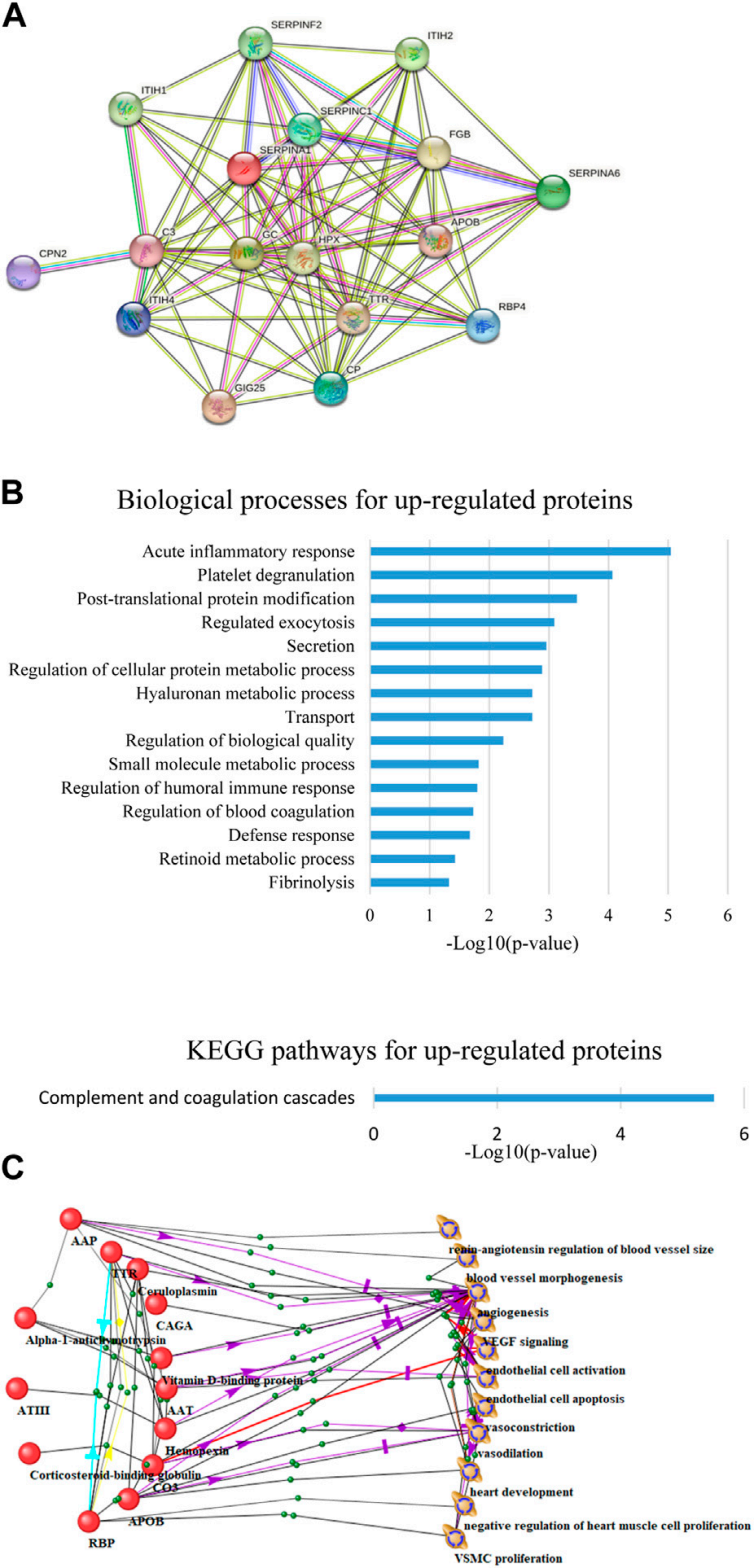
**(A)** Upregulated protein interaction network constructed using the STRING web resource. **(B)** Biological processes and KEGG pathways involving overexpressed proteins. **(C)** Connections of proteins significantly changed by the end of HDBR with the processes occurring in the cardiovascular system.

As a result of proteomic analysis, 533 different proteins were identified in plasma samples. Based on statistical analysis using the Mann-Whitney test (*p*-value <0.05), 25 proteins were identified whose levels changed significantly by the end of HDBR. Among the 17 proteins whose concentration increased by the end of the experiment (Figure 1A), there were proteins involved in the complement and coagulation cascade (C3, FGB, SERPINF2, SERPINC1, SERPINA1), acute phase response (ITIH4, SERPINF2, SERPINC1, GIG25, SERPINA1), platelet degranulation (ITIH4, FGB, SERPINF2, GIG25, SERPINA1), blood coagulation (FGB, SERPINF2, SERPINC1), fibrinolysis (FGB, SERPINF2), transport (APOB, TTR, C3, CP, HPX, ITIH4, FGB, SERPINF2, SERPINC1, RBP4, GIG25, SERPINA1, GC), retinoid metabolic processes (APOB, TTR, RBP4), etc. (Figure 1B).

It was found that eight proteins, the concentration of which significantly decreased by the end of HDBR, had various functions from mRNA splicing and cell cytoskeleton remodeling, to heme catabolism and oxygen transport (Table 1). However, processes and pathways connecting these proteins have not been identified.

**TABLE 1:** Functions of downregulated proteins.

Gene	Protein	Function
BPGM	Bisphosphoglycerate mutase	Regulates the affinity of hemoglobin to O2
SLU7	Pre-mRNA-splicing factor SLU7	mRNA splicing
CAPNS1	Calpain small subunit 1	Remodeling of the cytoskeleton
HBD	Hemoglobin subunit delta	Oxygen transport
S100A8	Protein S100-A8	Regulation of inflammatory responses
BLVRB	Flavin reductase	NADPH-dependent reduction of various flavins, heme catabolism
MYO1C	Unconventional myosin-Ic	Provides intracellular movement, including glucose transporters GLUT4 to the plasma membrane
UBE2V1	Ubiquitin-conjugating enzyme E2 variant 1	Plays a role in cell cycle control and differentiation

## 4 Discussion

### 4.1 Comparison of results with literature data

Our data are confirmed by previous studies of HDBR. Thus, it has been shown that there is an increase in the concentration of FGB (fibrinogen), ITIH2 and ITIH4 (inter-alpha-trypsin inhibitor heavy chains 2 and 4), and SerpinF2 (α2-antiplasmin) in HDBR (Kashirina et al., 2020). Although microgravity simulation experiments did not reveal activation of the coagulation cascade during the experiments (Haider et al., 2013; Cvirn et al., 2015). However, after 21 days of HDBR in the recovery period, there was an increase in the peak and rate of thrombin formation (Waha et al., 2015), which indicated an increased risk of thrombosis after the end of the experiment (Cvirn et al., 2015; Waha et al., 2015). Apparently, at the end of the experiment, increased hydrostatic pressure and shear stress in unadapted lower body vessels cause activation of blood coagulation mediated by the endothelial cell secretome (Cvirn et al., 2015). Therefore, the study of the proteomic composition of blood, which is in direct contact with endothelial cells, can shed light on the functional state of endothelial cells or reveal blood proteins that contributed to the balance shift toward procoagulation properties of the endothelium.

In this experiment, one of the possible reasons for the decrease of bisphosphoglycerate mutase (BPGM) and hemoglobin subunit delta (HBD) levels could be a decrease in red blood cell mass (RBCM), however, studies show that RBCM does not decrease in short bed rests (Branch et al., 1998) or decreases only after the end of the experiment (Leach and Johnson, 1984). Although there is evidence of a significant decrease in erythropoietin during HDBR (Gunga et al., 1996), which was observed in HDBR as early as the first few days, the effect of erythropoietin on RBCM could not have occurred in such a short time. Regarding long-duration spaceflight, the phenomenon of hemolysis is widely documented (Trudel et al., 2022), but no hemolysis was detected under conditions of short HDBR. On the contrary, HDBR caused hemoconcentration, and circulating parameters of hemolysis remained unchanged throughout bed rest (Trudel et al., 2017).

### 4.2 Association of up- and downregulated proteins with processes occurring in the CVS

Within the framework of this study, the relationships between significantly changed proteins and processes occurring in the CVS were analyzed using the ANDSystem program. This approach identified proteins directly or indirectly related to processes such as activation and apoptosis of endothelial cells, VEGF signaling, vasoconstriction, vasodilation, and angiogenesis (Figure 1C).

One of the proteins, RBP, retinol-binding protein 4, has been associated with processes directly related to the heart: negative regulation of heart muscle cell proliferation, heart muscle development (Figure 1C). RBP4 is an adipokine whose main function is retinol transport, but its association with cardiovascular disease (CVD) is now widely known: it correlates with increased thickness of the carotid intima-media complex, early onset of cardiovascular disease [Li et al., 2019 RBP4 is thought to mediate insulin-induced VSMC proliferation and contribute to the development of atherosclerosis (Li et al., 2014). The effect of RBP4 on the cardiovascular system may be mediated by endothelial cells, as carbachol-induced endothelium-dependent vasodilation of carotid arteries *ex vivo* was enhanced in RBP4 knockout mice and, conversely, reduced in RBP4 overexpressing mice (Kraus et al., 2015). Elevated levels of RBP4 attract macrophages to adipose tissue and promote local inflammation by causing macrophages to secrete pro-inflammatory cytokines: nuclear factor κ-B (NFκB), c-Jun N-terminal kinases (JNK), and interleukin 1β, leading to insulin resistance (Yang et al., 2005). The level of this protein decreased during exercise (60 min of bicycling and running per day for at least 3 days per week) (Graham et al., 2006). Moreover, serum RBP4 levels decreased in people in whom exercise improved insulin sensitivity, but not in those people in whom this effect was not achieved (Graham et al., 2006), indicating an RBP4-mediated link between a physically inactive lifestyle and insulin resistance. Moreover, studies in mice have shown that elevated serum levels of RBP4 can induce insulin resistance (Yang et al., 2005). Thus, accurate measurements of serum RBP4 levels can provide a quantitative measure of disease severity and guide the development of drugs to improve insulin sensitivity. In addition, since RBP4 levels are elevated in the serum of thin, non-diabetic but insulin-resistant individuals with a high genetic risk of developing type 2 diabetes (Graham et al., 2006), RBP4 may be useful as an early marker of diabetes risk.

Among other things, GLUT4 protein (the main insulin-stimulated glucose transporter) in adipocytes and serum RBP4 levels have been shown to be inversely correlated (Graham et al., 2006). Although we did not detect GLUT4 in the samples, nevertheless, by day 21 of HDBR, a significant decrease in the concentration of actin-associated molecular motor protein (MYO1C) was detected, which, as shown in the article (Toyoda et al., 2011), is able to regulate glucose uptake and improve insulin sensitivity. It is known, that HDBR conditions affect carbohydrate metabolism - insulin secretion increases and impaired glucose tolerance develops (Afonin, 1989). Insulin resistance can cause endothelial dysfunction, which is dangerous for the development of cardiovascular diseases. Thus, during HDBR the level of RBP4 increased, altering insulin sensitivity and modulating the molecular interrelations of other cardiovascular risk factors.

Apolipoprotein B (APOB), which is the only apolipoprotein of low-density lipoproteins that causes cholesterol accumulation in vessel walls, also plays an important role in the regulation of endothelial function. It has been demonstrated that apolipoprotein B impairs endothelium-dependent vasodilation with attenuation of NO-mediated pathway efficiency (Zhang et al., 2014). This agrees well with the results of a large cohort study of 1,016 subjects which showed that the level of apolipoprotein B is inversely correlated with endothelium-dependent vasodilation (Lind, 2007).

The link with cardiovascular processes is also demonstrated by complement component C3, which plays a central role in all three pathways of complement system activation: classical, alternative and lectin. The endothelial monolayer of all blood vessels, due to its location, is in close contact with the circulating complement components. Some of the complement factors can directly or indirectly affect the endothelium (Hertle et al., 2014). Endothelial cells express anaphylatoxin receptors and complement regulators on their surface, suggesting that they are a direct target of complement molecules (Propson et al., 2021; Schraufstatter et al., 2002). C3a, C5a, and C5b have been shown to induce the expression of adhesion molecules and pro-inflammatory cytokines in endothelial cells *in vitro* (Bossi et al., 2011), which initiates and promotes the development of atherosclerosis. It should be noted that proteins of the complement system had the highest representation among the proteins specific for 21 days of HDBR, confirming the involvement of the complement system in response to bed rest conditions.

## 5 Conclusion

**FIGURE 2:**
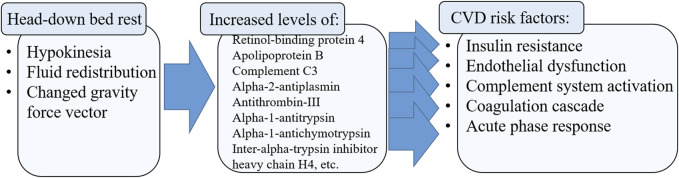
Schematic presentation of results in the context of human health.

HDBR conditions led to an increase in the levels of proteins involved in the complement system and the coagulation cascade, platelet degranulation, fibrinolysis, acute phase proteins, post-translational modification of proteins and other proteins that affect the functions of endothelial cells (Figure 2). At the same time, the levels of proteins involved in amino acid biosynthesis, glycolysis, oxygen transport, heme catabolism, etc., decreased in the blood of healthy volunteers.

Thus, on day 21 of HDBR, the levels of RBP4, APOB and C3 proteins increased, each of which plays an important role in one way or another in modulating the properties of the endothelium and contributing to the development of atherosclerosis, which is a precursor of many cardiovascular diseases. Identification of the molecular mechanisms of changes in vascular endothelial function may help to develop preventive measures to counteract the negative effects of prolonged hypokinesia. And although 21 days of HDBR is not enough for the development of serious clinically significant changes in the activity of the cardiovascular system, we can still capture the first molecular reactants to the conditions of hypodynamia and hypokinesia, which may trigger subsequent processes associated with deterioration in the activity of blood vessels and the heart, which can no longer be reversed. It is therefore particularly important to identify early signs of changes in vascular function that can still be compensated for.

## 6 Limitations

Finally, the limitations of this work should be noted. The main physiological mechanisms that trigger changes in the blood proteomic profile are associated with thoracocranial redistribution of blood (Millet et al., 2001), changes in the regulation of vascular tone (Rusanov et al., 2020), a decrease in cardiac output under conditions of HDBR (Greaves et al., 2019). However, this study did not include an instrumental study of the above indicators. Another limitation of the work is the study of a small sample of subjects, but such complex human studies are mostly carried out on a small sample and provide unique results of theoretical and practical importance.

## Data Availability

The datasets presented in this study can be found in online repositories. The names of the repository/repositories and accession number(s) can be found below: http://www.proteomexchange.org/, identifier PXD053149.
